# The rise of ChatGPT-4: exploring its efficacy as a decision support tool in esophageal surgery – a research letter

**DOI:** 10.1097/JS9.0000000000001696

**Published:** 2024-05-29

**Authors:** Jianfeng Zhou, Yixin Liu, Yushang Yang, Pinhao Fang, Longqi Chen, Yong Yuan

**Affiliations:** Department of Thoracic Surgery and Institute of Thoracic Oncology, West China Hospital of Sichuan University, Chengdu, Sichuan, People’s Republic of China

*Dear Editor*,

We recently read with great interest the article titled ‘Evaluation of Large Language Models in Breast Cancer Clinical Scenarios’ by Deng *et al*. published in the *International Journal of Surgery.* ^1^ The authors provided a comprehensive analysis comparing the performance of ChatGPT-3.5, ChatGPT-4.0, and Claude2 in various clinical scenarios related to breast cancer. The study’s findings revealed that in the realm of clinical applications for breast cancer, GPT-4.0 showcases not only superiority in terms of quality and relevance but also demonstrates exceptional capability in applicability, when compared to GPT-3.5 and Claude2. Our own research, detailed in ‘The Rise of ChatGPT-4: Exploring its Efficacy as a Decision Support Tool in Esophageal Surgery’, shares similar methodologies and conclusions, emphasizing the potential of Large Language Models like ChatGPT-4 in esophageal surgery applications.

In 2022, the groundbreaking Chat Generative Pre-trained Transformer (Chat-GPT) was introduced as a versatile language model with diverse applications^2^. Within the medical sphere, Chat-GPT has exhibited remarkable potential^3–5^: Zhu *et al*.^3^ evaluated Chat-GPT’s responses to fundamental cardiovascular disease prevention inquiries, and its accuracy rate are 96% and 92.1% for the American Heart Association Basic Life Support and Advanced Cardiovascular Life Support exams, respectively. In a separate investigation, Yee *et al*.^5^ demonstrated Chat-GPT’s superiority, regurgitated extensive knowledge of cirrhosis (79.1% correct) and hepatocellular carcinoma (74.0% correct). Our study is dedicated to exploring the prospective integration of Chat-GPT4 in Esophageal Surgery, with a specific focus on the four major esophageal disorders (esophageal cancer, Barrett’s esophagus, gastroesophageal reflux disease, achalasia of cardia) as illustrative cases. We aim to dissect surgical indications, patient adaptation, perioperative planning, and postoperative follow-up, along with other pivotal considerations.

We conducted an inquiry comprising 35 questions concerning esophageal cancer, Barrett’s esophagus, gastroesophageal reflux disease, and achalasia of cardia. The responses were sourced from Chat-GPT4 (Version: Nov 2023) and can be found in the Supplementary Material (Supplemental Digital Content 1, http://links.lww.com/JS9/C676). Each response was subjected to rigorous evaluation by three Esophageal Surgery Specialists. They applied guidelines from both the International Society for Diseases of the Esophagus (ISDE) and the National Comprehensive Cancer Network (NCCN), alongside their own extensive clinical experience. To appraise the responses, a scoring system was employed, ranging from 0 to 3. A score of 0 signified complete noncompliance or irrelevance, while a score of 3 indicated nearly impeccable alignment with clinical guidelines and practices, offering robust guidance value. Recognizing the inherent subjectivity in scoring, the ultimate assessment of Chat-GPT4’s responses hinged on the median score conferred by the three experts. It is noteworthy that ChatGPT can enhance its performance through data input, an essential process for augmenting the model’s functionality. GPT-4 has the ability to accumulate knowledge via internet searches. Therefore, we clear GPT-4’s memory after each query to mitigate potential biases that may be introduced in practice.

Out of the 35 responses, merely one (2.8%) received a score of 1, with 10 (28.5%) attaining a score of 2, and the majority, 24 (68.5%), securing a score of 3 (Table 1). The experts were remarkably aligned, with no score deviating by more than a single point between any two experts for any given response. This robustly suggests that in nearly all instances (97.2%), Chat-GPT4 furnishes commendable responses (Fig. 1). In instances where a score of 1 was assigned, Chat-GPT4 recommends follow-up procedures for asymptomatic patients after esophageal cancer surgery entailing a thorough history and comprehensive evaluation every 3–6 months for the initial 2 years, followed by adjustments to every 6–12 months for 3 to 5 years, then once a year. Notably, the NCCN guidelines (from Version 1.2023) have omitted the inclusion of ‘then once a year’. It is crucial to recognize that a staggering 90% of recurrences manifest within the first 2 years postlocal treatment completion, which diminishes the necessity for routine surveillance after 5 years. While this response is not entirely incorrect, its guidance may have a degree of constraint. Depending on risk factors and comorbidities, the consideration of additional follow-up after 5 years may be warranted.

**Table 1:** Scores for responses from Chat-GPT4.

	Scores^a^			
Questions	Expert 1	Expert 2	Expert 3	Median
1.Before esophageal surgery, how do you assess a patient’s nutritional status, and what steps would you take if malnutrition is detected?	3	2	2	2
2.What is the role of neoadjuvant therapy in the treatment plan for esophageal cancer, and how does it impact surgical decision-making?	2	3	3	3
3.Can you explain the different surgical approaches (e.g. Ivor-Lewis, McKeown, transhiatal) for esophagectomy and their respective indications?	3	3	3	3
4.How do you decide between open and minimally invasive approaches for esophageal surgery, and what are the associated benefits and risks?	2	3	2	2
5.Outline the crucial factors to take into account for lymphadenectomy during esophagectomy, and elucidate how the scope of lymph node dissection impacts prognosis.	2	3	3	3
6.Discuss your approach to handling intraoperative challenges, including the identification of the recurrent laryngeal nerve and the prevention of adjacent structure injuries.	3	2	3	3
7.Explain the methods employed to guarantee sufficient blood supply to the gastric conduit during esophagectomy.	2	3	3	3
8.Discuss your approach to handling intraoperative complications such as bleeding or perforation during esophageal surgery.	2	3	2	2
9.What are the options for esophageal reconstruction after esophagectomy, and how do you choose between gastric pull-up, colonic interposition, or other techniques?	3	2	2	2
10.What is the frequency of follow-up for asymptomatic patients after esophageal cancer surgery	1	1	2	1
11.Explain your approach to managing delayed gastric emptying or dumping syndrome in patients with reconstructed gastric conduits.	3	3	2	3
12.Discuss the criteria and methodologies for endoscopic surveillance postesophageal surgery, particularly in cases involving Barrett’s esophagus.	3	3	3	3
13.Explain your approach to dealing with postoperative esophageal stricture formation, including the available treatment modalities.	3	3	3	3
14.Discuss your strategy for managing anastomotic leaks and the potential implications for long-term outcomes.	2	3	3	3
15.What is the role of adjuvant therapy in patients who have undergone surgical resection of esophageal cancer, and how does it influence follow-up care?	2	3	3	3
16.Outline your protocol for evaluating and addressing postoperative complications such as pulmonary embolism or deep vein thrombosis in patients undergoing esophageal surgery.	2	2	3	2
17.Share the recommended approach to the management of patients who experience recurrent or metastatic esophageal cancer following their initial surgical resection.	2	3	3	3
18.How do you address nutritional support in the postoperative period for patients who have undergone esophageal surgery?	2	2	3	2
19.Discuss the approaches to addressing postoperative gastroesophageal reflux in patients who have undergone esophagectomy.	3	3	2	3
20.What is the target intraoperative blood loss volume, based on the patient’s preoperative hemoglobin level and comorbidities, to minimize the risk of postoperative complications and ensure adequate tissue oxygenation during esophagectomy?	3	3	3	3
21.Elaborate on the role of minimally invasive techniques, such as robotic surgery, in esophageal procedures, and highlight the potential advantages over traditional open approaches.	2	2	2	2
22.Explain your approach to managing complications associated with conduit ischemia or necrosis following esophagectomy.	2	2	3	2
23.Discuss the pivotal factors in the surgical treatment of achalasia, and elaborate on how the selection of a procedure (e.g. Heller myotomy) influences outcomes.	3	3	3	3
24.How do you approach patients with advanced esophageal cancer who are not candidates for curative surgery, and what are the palliative options available?	2	3	3	3
25.Outline the criteria for considering salvage surgery in instances of recurrent esophageal cancer following initial treatment.	3	3	3	3
26.How do you assess and manage postoperative infections in patients who have undergone esophageal surgery?	3	3	3	3
27.Elaborate on the long-term quality of life outcomes for patients following esophageal surgery, and delineate how these outcomes may differ based on the surgical approach and type of disease.	2	3	3	3
28.How do you address postoperative psychological and emotional well-being in patients who have undergone esophageal surgery, particularly those with a history of mental disorders?	2	2	3	2
29.Outline the factors to be taken into account for preserving fertility in young patients undergoing esophageal surgery.	3	2	3	3
30.Discuss the criteria used to assess the suitability of esophageal surgery for elderly patients with esophageal disease, and highlight the particular perioperative factors taken into account.	3	3	3	3
31.A 65-year-old male presents with a T1N0 adenocarcinoma at the gastroesophageal junction. What are the considerations for choosing between endoscopic resection and surgical intervention? How might patient factors influence this decision?	3	3	3	3
32.A 50-year-old female with longstanding achalasia has failed conservative treatment. What are the advantages and potential complications of different surgical approaches for treating achalasia? How do you select the most appropriate technique for this patient?	3	3	3	3
33.A 60-year-old male with a history of esophagectomy for cancer now presents with recurrent dysphagia. What diagnostic evaluations and interventions would you consider to evaluate the cause of his symptoms?	2	2	2	2
34.A 70-year-old patient develops a chyle leak postesophagectomy. What are the immediate steps for management, and what are the potential long-term consequences of this complication?	3	3	3	3
35.55-year-old male develops an anastomotic stricture following esophagectomy. How would you approach the treatment of this stricture, and what are the considerations for long-term management?	2	3	3	3

a Each response was subjected to rigorous evaluation by three Esophageal Surgery Specialists based on guidelines from both the International Society for Diseases of the Esophagus (ISDE) and the National Comprehensive Cancer Network (NCCN), alongside their own extensive clinical experience. The scoring system ranges from 0 to 3, with 0, 1, 2, and 3 representing the following: complete noncompliance with clinical guidelines and practices or irrelevant; less than 50% compliance with clinical guidelines and practices, limited guidance value; more than 50% compliance with clinical guidelines and practices, offering some guidance value; nearly complete compliance with clinical guidelines and practices, providing strong guidance value.

**Figure 1:**
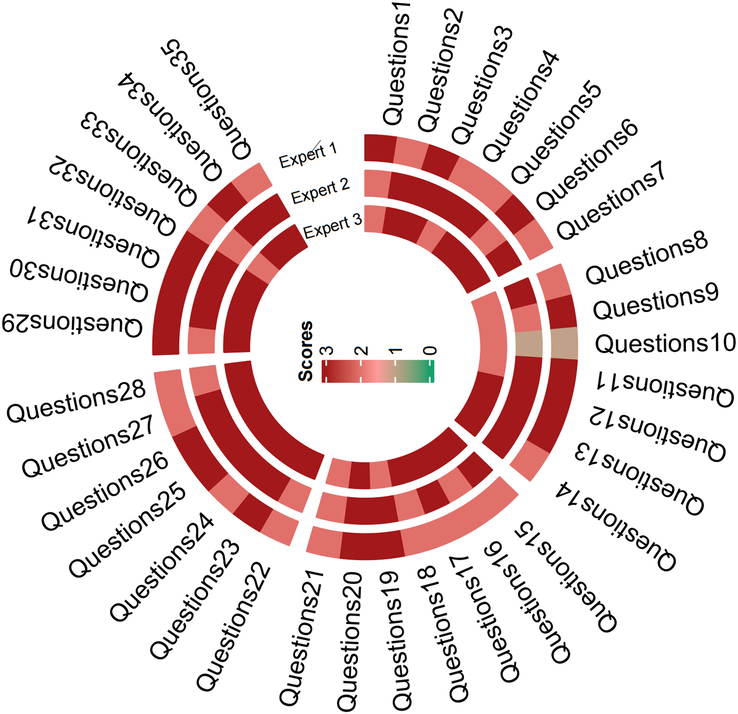
Scores for responses from Chat-GPT4 of three experts. Each region corresponds to different questions. Circles from outside are the score for Chat-GPT4 responses of three experts. Dark red represents a score of 3, light red represents a score of 2, and light brown represents a score of 1.

The burden of esophageal diseases significantly impacts global healthcare, necessitating innovative solutions to enhance patient outcomes^6^. The integration of machine learning into surgical practice heralds an era of transformation in the healthcare sector^7,8^, with tools like ChatGPT possessing the potential to support esophageal surgery decision-making. In a nutshell, our study underscores Chat-GPT4’s commendable ability to address a majority of inquiries in the realm of Esophageal Surgery, marking it as a promising tool in the field of esophageal surgeon. While it is acknowledged that responses generated by ChatGPT can often be found through internet searches, the value of integrating such an AI tool in clinical practice lies in its capability to rapidly synthesize and contextualize vast amounts of medical data, providing immediate support in decision-making processes. However, it is imperative to acknowledge the constraints that come with Chat-GPT4. Firstly, Chat-GPT does not have access to medical textbooks, journals, or any real-time medical databases, while Chat-GPT can generate coherent responses, it may not fully understand the clinical context, subtleties, or nuances involved in complex surgical procedures. In addition, Chat-GPT communicates exclusively through text. It cannot completely observe a surgical procedure, collaborate with a surgical team, or physically interact with a patient. Moreover, Chat-GPT cannot substitute for the empathy, detailed communication, and care provided by healthcare professionals. The indispensability of human warmth, empathy, and the ability to understand and respond to nonverbal cues in patient care is irreplaceable. Furthermore, the incident involving the erroneous ‘then once a year’ follow-up recommendation underscores the indispensable role of human oversight, reinforcing the need for AI tools to complement, not replace, the clinical acumen of medical professionals. Considering these nuances, while Chat-GPT4 stands as a reasonably proficient resource in the domain of esophageal surgery, it is important to recognize potential limitations in its ability to provide optimal assistance to cancer patients.

## Ethical approval

This analysis of publicly available data does not require ethical approval.

## Source of funding

This work was supported by the National Natural Science Foundation of China (81970481, 82000514), National Natural Science Foundation Regional Innovation and Development (U20A20394), Sichuan Science and Technology Program (2022YFS0048, 2021YFS0222), 1.3.5 project for disciplines of excellence, West China Hospital, Sichuan University (2020HXFH047, ZYJC18010 and 20HXJS005, 2018HXFH020), and China Postdoctoral Science Foundation (2020M673241).

## Author contribution

J.Z. and Y.L. (Conceptualization, Data curation, Writing – original draft, Writing – review and editing), Y.Y. (Conceptualization, Writing – original draft, Writing – review and editing), P.F. (Writing – review and editing), L.C. (Conceptualization, Writing – review and editing), and Y.Y. (Conceptualization, Writing – original draft, Writing – review and editing).

## Conflicts of interest disclosure

The authors declare that the research was conducted in the absence of any commercial or financial relationships that could be construed as a potential conflict of interest.

## Data availability statement

All data are included in the manuscript.

## Supplementary Material

## References

[1] Deng L Wang T Yangzhang . Evaluation of large language models in breast cancer clinical scenarios: a comparative analysis based on ChatGPT-3.5, ChatGPT-4.0, and Claude2. Int J Surg 2024;110:1941–1950. 38668655 10.1097/JS9.0000000000001066 PMC11019981

[2] Petrić Howe N Skipper M Van Noorden R . Nature’s take: how will ChatGPT and generative AI transform research? Nature 2023. doi:10.1038/d41586-023-03467-8 37923951

[3] Zhu L Mou W Yang T . ChatGPT can pass the AHA exams: open-ended questions outperform multiple-choice format. Resuscitation 2023;188:109783. 37349064 10.1016/j.resuscitation.2023.109783

[4] Vela Ulloa J King Valenzuela S Riquoir Altamirano C . Artificial intelligence-based decision-making: can ChatGPT replace a multidisciplinary tumour board? Br J Surg 2023;110:1543–1544. 37595064 10.1093/bjs/znad264

[5] Yeo YH Samaan JS Ng WH . Assessing the performance of ChatGPT in answering questions regarding cirrhosis and hepatocellular carcinoma. Clin Mol Hepatol 2023;29:721–732. 36946005 10.3350/cmh.2023.0089 PMC10366809

[6] Luan S Xie R Yang Y . Acid-responsive aggregated gold nanoparticles for radiosensitization and synergistic chemoradiotherapy in the treatment of esophageal cancer. Small 2022;18:e2200115. 35261151 10.1002/smll.202200115

[7] Li B Feridooni T Cuen-Ojeda C . Machine learning in vascular surgery: a systematic review and critical appraisal. NPJ Digit Med 2022;5:7. 35046493 10.1038/s41746-021-00552-y PMC8770468

[8] Marwaha JS Raza MM Kvedar JC . The digital transformation of surgery. NPJ Digit Med 2023;6:103. 37258642 10.1038/s41746-023-00846-3 PMC10232406

