# Supplementary material for: The rise of ChatGPT-4: exploring its efficacy as a decision support tool in esophageal surgery – a research letter
Source: Int J Surg. 2024 May 29;110(9):5928–30. doi: 10.1097/JS9.0000000000001696 (PMC11392133; doi:10.1097/JS9.0000000000001696)
Supplement: Supplementary file 1 [file js9-110-5928-s001.pdf]

The meaning of each score:
-0: Complete non-compliance with clinical guidelines and practices or irrelevant.
-1: less than 50% compliance with clinical guidelines and practices, limited guidance value.
-2: more than 50% compliance with clinical guidelines and practices, offering some guidance value.
-3: nearly complete compliance with clinical guidelines and practices, providing strong guidance value.

1. Before esophageal surgery, how do you assess a patient's nutritional status, and what steps would you take if malnutrition is detected?

Answer1.1: In a patient scheduled for esophagectomy, we assess nutritional status through a combination of subjective and objective measures. This includes a thorough dietary history, assessment of weight changes, serum albumin and prealbumin levels, and potentially more detailed assessments like body composition analysis. If malnutrition is identified, interventions may range from dietary modification and oral supplements to more aggressive interventions like enteral or parenteral nutrition support.

Answer1.2: Before esophageal surgery, assessing a patient's nutritional status involves evaluating weight history, dietary intake, biochemical markers (e.g., albumin, prealbumin), and physical findings (e.g., muscle wasting). If malnutrition is detected, a multidisciplinary approach is initiated, including nutritional supplementation (oral, enteral, or parenteral nutrition) and possibly delaying surgery to optimize nutritional status, thereby reducing perioperative risks.

Answer1.3: Evaluate nutritional status using body mass index (BMI), serum protein levels (albumin and prealbumin), and dietary intake history. Nutritional risk screening tools like the Nutritional Risk Screening (NRS-2002) or the Malnutrition Universal Screening Tool (MUST) can be utilized. If malnutrition is detected, initiate nutritional support early. This may involve oral nutritional supplements, enteral nutrition via a nasogastric tube or a percutaneous endoscopic gastrostomy (PEG), or parenteral nutrition if enteral routes are not feasible.

☐ 0 ☐ 1 ☐ 2 ☐ 3

2. What is the role of neoadjuvant therapy in the treatment plan for esophageal cancer, and how does it impact surgical decision-making?

Answer1.1: Consider a patient with locally advanced esophageal cancer (Stage II/III). Neoadjuvant therapy, typically a combination of chemotherapy and radiation, is administered before surgery. This aims to shrink the tumor and potentially downstage it, making surgical resection more feasible and effective. The response to neoadjuvant therapy influences the extent of surgery and nodal dissection required.

Answer1.2: Neoadjuvant therapy, which includes chemotherapy and/or radiotherapy before surgery, aims to reduce tumor size, address micrometastases, and improve surgical outcomes. Its role in the treatment plan is significant for locally advanced esophageal cancer, as it can increase the chance of complete resection and potentially

improve survival. This therapy impacts surgical decision-making by potentially altering the extent of surgery required and by influencing the timing of surgery.

Answer1.3: Neoadjuvant therapy (chemotherapy, radiotherapy, or a combination of both) is often used before surgery to shrink the tumor, making it easier to remove and potentially improving survival rates. It also targets micrometastases early. The impact on surgical decision-making includes altering the stage of the disease and possibly converting an unresectable tumor into a resectable one.

☐ 0 ☐ 1 ☐ 2 ☐ 3

3. Can you explain the different surgical approaches (e.g., Ivor-Lewis, McKeown, transhiatal) for esophagectomy and their respective indications?

Answer1.1: Let's take the example of a patient with lower esophageal cancer. The choice of surgical approach depends on factors like tumor location, extent, and patient characteristics. For instance, an Ivor-Lewis esophagectomy may be preferred for tumors in the mid-to-lower esophagus, while a McKeown approach might be chosen for more proximal tumors. A transhiatal approach, on the other hand, may be considered for selected cases with distal esophageal or gastroesophageal junction tumors.

Answer1.2: The Ivor-Lewis (thoracoabdominal), McKeown (three-field), and transhiatal approaches are the primary surgical techniques for esophagectomy. The choice among them depends on tumor location, extent, patient's anatomy, and surgeon's expertise. Ivor-Lewis is preferred for tumors in the lower esophagus, McKeown for higher tumors requiring extensive lymphadenectomy, and transhiatal for patients with comorbidities making thoracotomy high risk.

Answer1.3: Ivor-Lewis Esophagectomy: Indicated for tumors of the mid to distal esophagus, involves a right thoracotomy and abdominal incision for gastric mobilization and anastomosis in the chest; McKeown Esophagectomy: Suitable for tumors of the upper esophagus or gastroesophageal junction, includes a right thoracotomy, laparotomy, and neck incision for cervical anastomosis; Transhiatal Esophagectomy: For distal esophageal cancers, performed without thoracotomy, involving abdominal and neck incisions. Less respiratory complications but higher risk of recurrent laryngeal nerve injury.

☐ 0 ☐ 1 ☐ 2 ☐ 3

4. How do you decide between open and minimally invasive approaches for esophageal surgery, and what are the associated benefits and risks?

Answer1.1: Consider a patient with early-stage esophageal cancer. Minimally invasive esophagectomy (MIE) offers potential advantages like reduced blood loss, shorter hospital stay, and faster recovery compared to open surgery. However, patient factors like obesity or prior abdominal surgeries may influence the decision. Additionally, the surgeon's experience and expertise with minimally invasive techniques play a crucial role in the decision-making process.

Answer1.2: The choice between open and minimally invasive approaches (laparoscopic or robotic) depends on the patient's overall health, the stage and location

of the tumor, and the surgeon's experience. Minimally invasive surgery is associated with reduced postoperative pain, shorter hospital stays, and quicker recovery but requires specialized skills. The risks include potential for technical complications and the need for conversion to open surgery in some cases.

Answer1.3: Decision-making involves evaluating the tumor's location, patient's overall health, and surgeon's expertise. Minimally invasive surgery (MIS) offers benefits like reduced pain, shorter hospital stay, and quicker recovery. However, it requires significant surgical skill and may not be suitable for all tumors. Open surgery is preferred for large or complex tumors.

☐ 0 ☐ 1 ☐ 2 ☐ 3

5. Outline the crucial factors to take into account for lymphadenectomy during esophagectomy, and elucidate how the scope of lymph node dissection impacts prognosis.

Answer1.1: In a patient with esophageal cancer, a meticulous lymphadenectomy is crucial for accurate staging and potentially improving long-term outcomes. The extent of lymph node dissection depends on factors like tumor location and type. For example, in adenocarcinoma of the distal esophagus, a more extensive lymphadenectomy around the celiac axis and left gastric artery may be performed compared to squamous cell carcinoma located in the mid-esophagus.

Answer1.2: The extent of lymph node dissection is a critical factor, with more extensive dissection potentially improving staging accuracy and survival but increasing the risk of complications. The decision on the scope of lymphadenectomy is influenced by tumor location, stage, and the balance between oncological benefit and risk of morbidity.

Answer1.3: The extent of lymph node dissection can influence staging accuracy and prognosis. Factors include tumor location, depth of invasion, and histological type. A more extensive lymphadenectomy may improve survival by ensuring removal of potential metastatic nodes but increases the risk of complications.

☐ 0 ☐ 1 ☐ 2 ☐ 3

6. Discuss your approach to handling intraoperative challenges, including the identification of the recurrent laryngeal nerve and the prevention of adjacent structure injuries.

Answer1.1: During an esophagectomy, it's critical to identify and preserve the recurrent laryngeal nerve to maintain vocal cord function. Careful dissection and meticulous attention to anatomical landmarks, aided by intraoperative nerve monitoring, can help in identifying and preserving the nerve. Additionally, avoiding injury to adjacent structures like the trachea or aorta requires a thorough understanding of the surgical anatomy.

Answer1.2: Handling intraoperative challenges involves meticulous surgical technique to identify and protect the recurrent laryngeal nerve, prevent injury to adjacent structures, and ensure clear surgical margins. Strategies include using intraoperative nerve monitoring and adopting careful dissection techniques.

Answer1.3: Careful surgical technique to identify and protect the recurrent laryngeal nerve is crucial. Use of intraoperative nerve monitoring may reduce injury risk. Prevention of injury to adjacent structures requires thorough anatomical knowledge and careful dissection.

☐ 0 ☐ 1 ☐ 2 ☐ 3

7. Explain the methods employed to guarantee sufficient blood supply to the gastric conduit during esophagectomy.

Answer1.1: In creating a gastric conduit for reconstruction, preserving the blood supply is crucial to prevent ischemia or necrosis. This can be achieved by careful dissection of the right gastroepiploic vessels, maintaining an adequate length of the pedicle, and avoiding excessive tension on the conduit during the anastomosis.

Answer1.2: Techniques to ensure sufficient blood supply to the gastric conduit include preserving the right gastric and gastroepiploic arteries during dissection and performing intraoperative assessments of vascular perfusion, such as using indocyanine green fluorescence imaging.

Answer1.3: Techniques include careful dissection to preserve the right gastroepiploic artery and vein, assessment of gastric conduit perfusion using intraoperative fluorescence angiography, and avoiding tension on the gastric conduit.

☐ 0 ☐ 1 ☐ 2 ☐ 3

8. Discuss your approach to handling intraoperative complications such as bleeding or perforation during esophageal surgery.

Answer1.1: If intraoperative bleeding occurs, measures like meticulous hemostasis, temporary vascular clamping, and potential conversion to an open procedure may be necessary. In cases of perforation, immediate repair or reinforcement of the defect using tissue flaps or synthetic materials may be performed to prevent postoperative complications.

Answer1.2: Immediate management of complications like bleeding involves controlling the source of hemorrhage, possibly requiring additional incisions or conversion to open surgery. For perforation, repair and reinforcement of the suture line may be necessary, sometimes with protective stenting.

Answer1.3: Immediate control of bleeding using surgical techniques and hemostatic agents. For perforation, immediate repair and reinforcement of the repair site are critical. Preparedness and rapid response are key to managing these complications effectively.

☐ 0 ☐ 1 ☐ 2 ☐ 3

9. What are the options for esophageal reconstruction after esophagectomy, and how do you choose between gastric pull-up, colonic interposition, or other techniques?

Answer1.1: The choice of reconstruction technique depends on various factors, including patient anatomy, comorbidities, and surgeon preference. For instance, a gastric pull-up may be preferred for its proximity and vascular supply, but colonic interposition may be chosen in cases where the stomach is not suitable or if there are

concerns about reflux. Each technique has its own advantages and potential drawbacks.

Answer1.2: The choice between gastric pull-up, colonic interposition, and other techniques depends on factors like the length of the esophagus that needs to be replaced, previous surgeries, and patient-specific considerations. Gastric pull-up is most common, with colonic interposition used when the stomach is not viable.

Answer1.3: Gastric Pull-up: Most common technique due to its simplicity and reliability.Colonic Interposition: Used when the stomach is not viable for use or has been previously resected.Selection is based on the individual patient's anatomy, previous surgeries, and the condition of potential conduit organs.

☐ 0 ☐ 1 ☐ 2 ☐ 3

10. What is the frequency of follow-up for asymptomatic patients after esophageal cancer surgery

Answer1.1: Follow-up Interval: Every 3-6 months for the first 2 years, then every 6-12 months for the next 3 years, and annually thereafter. A patient with esophageal cancer who underwent curative surgery would typically have more frequent follow-up appointments in the initial years to closely monitor for any signs of recurrence. After the first few years, follow-up visits become less frequent.

Answer1.2: For asymptomatic patients, follow-up typically includes clinical assessment, imaging, and endoscopy at intervals that become less frequent over time, usually starting every 3-6 months for the first two years, then annually.

Answer1.3: Typically, follow-up includes visits every 3-6 months for the first 2 years, then every 6-12 months up to 5 years, then annually. Imaging and endoscopic evaluations are part of follow-up to detect recurrences early.

☐ 0 ☐ 1 ☐ 2 ☐ 3

11. Explain your approach to managing delayed gastric emptying or dumping syndrome in patients with reconstructed gastric conduits.

Answer1.1: If a patient experiences delayed gastric emptying after esophagectomy and gastric conduit reconstruction, dietary modifications (small, frequent meals) and prokinetic medications may be prescribed. For dumping syndrome, which can occur due to rapid emptying of the gastric conduit, dietary adjustments (avoiding high sugar foods) and antispasmodic medications may be recommended.

Answer1.2: For delayed gastric emptying, dietary modifications (e.g., smaller, more frequent meals, avoidance of high-fat and high-fiber foods) and medications like prokinetics (e.g., metoclopramide) can be beneficial. For dumping syndrome, dietary adjustments (e.g., eating smaller, frequent meals, avoiding simple sugars, increasing protein and fiber intake) and medications to slow gastric emptying or reduce symptoms are recommended. Severe cases might require surgical revision.

Answer1.3: Initial management focuses on dietary modifications—small, frequent meals, avoiding simple sugars, and increasing protein and fiber intake to slow gastric emptying. Medications like octreotide for severe dumping syndrome and prokinetics for delayed gastric emptying can be effective. In refractory cases, surgical

interventions may be considered, such as reconstruction of the pylorus (pyloroplasty) or revision of the gastric conduit.

☐ 0 ☐ 1 ☐ 2 ☐ 3

12. Discuss the criteria and methodologies for endoscopic surveillance post-esophageal surgery, particularly in cases involving Barrett's esophagus.

Answer1.1: In a patient with a history of Barrett's esophagus who has undergone esophagectomy, surveillance endoscopy is indicated to monitor for any remaining or recurrent Barrett's mucosa in the gastric conduit. This is typically performed annually, and biopsies are taken as necessary. The technique involves careful examination of the gastric conduit and the anastomotic site.

Answer1.2: Surveillance is recommended for detecting recurrence or progression of Barrett's esophagus. The initial endoscopy is typically performed 6-12 months post-surgery, followed by periodic surveillance based on initial findings. High-resolution endoscopy with systematic biopsies from the anastomotic site and any residual Barrett's epithelium is standard.

Answer1.3: Surveillance is recommended for patients with a history of Barrett's esophagus or high-grade dysplasia treated surgically. The initial endoscopy should be performed 6 to 12 months post-surgery, followed by annual endoscopies if no dysplasia is detected. Biopsies should be taken from the anastomotic site and any residual Barrett's epithelium to monitor for dysplasia or carcinoma.

☐ 0 ☐ 1 ☐ 2 ☐ 3

13. Explain your approach to dealing with postoperative esophageal stricture formation, including the available treatment modalities.

Answer1.1: If a patient develops an anastomotic stricture post-esophageal surgery, treatment options may include endoscopic dilation, typically performed using a controlled radial expansion balloon or bougie dilator. In refractory cases, stenting or incisional therapy (like endoscopic incisional therapy with needle knife) may be considered. Regular surveillance endoscopy helps monitor and manage strictures.

Answer1.2: Endoscopic dilation is the primary treatment for strictures, with options ranging from balloon to bougie dilators. Steroid injections or temporary stent placement may be considered for refractory strictures. Regular follow-ups are necessary to evaluate symptom improvement and need for further interventions.

Answer1.3: Treatment typically starts with endoscopic dilation using balloons or bougies, often requiring multiple sessions. In cases of refractory or recurrent strictures, stent placement or surgical revision may be necessary. Injecting steroids into the stricture or applying mitomycin C topically can reduce the need for repeated dilations.

☐ 0 ☐ 1 ☐ 2 ☐ 3

14. Discuss your strategy for managing anastomotic leaks and the potential implications for long-term outcomes.

Answer1.1: In the event of an anastomotic leak after esophageal surgery, treatment involves a multidisciplinary approach. This may include drainage of the leak,

intravenous antibiotics, and nutritional support. In severe cases, reoperation may be necessary. Consequences of leaks can vary, ranging from minor delays in healing to more significant complications like abscess formation or mediastinitis, which can have long-term implications for the patient's recovery.

Answer1.2: Management includes nutritional support, broad-spectrum antibiotics, and drainage of any collections. Endoscopic interventions like stenting or clipping can be considered for smaller leaks. The implications for long-term outcomes depend on the severity of the leak and the success of initial management strategies, influencing recovery and potential for stricture formation.

Answer1.3: Management depends on the size of the leak and the patient's condition. Small, contained leaks may be managed conservatively with drainage, antibiotics, and nutritional support. Larger leaks or those associated with sepsis may require surgical intervention for repair or drainage. Long-term outcomes depend on early detection and effective management to prevent severe sepsis and organ failure.

☐ 0 ☐ 1 ☐ 2 ☐ 3

15. What is the role of adjuvant therapy in patients who have undergone surgical resection of esophageal cancer, and how does it influence follow-up care?

Answer1.1: Adjuvant therapy, typically chemotherapy or chemoradiotherapy, may be recommended in cases where the pathology report after surgery reveals adverse features like positive margins or extensive lymph node involvement. This additional treatment aims to reduce the risk of recurrence. Adjuvant therapy impacts follow-up care by necessitating close monitoring for potential side effects and toxicity, as well as ongoing surveillance for recurrence.

Answer1.2: Role and Influence on Follow-up: Adjuvant therapy (chemotherapy, radiotherapy, or both) is considered based on pathological findings post-resection. It aims to eliminate residual microscopic disease and reduce recurrence risk. Follow-up care includes monitoring for side effects and disease recurrence through clinical assessments and imaging.

Answer1.3: Role and Follow-up Care: Adjuvant therapy (chemotherapy, radiation, or both) is considered based on pathological findings post-resection, especially in cases with positive margins or lymph node involvement. It aims to eradicate residual disease and reduce recurrence risk. Follow-up care includes monitoring for toxicity, recurrence, and managing long-term effects of treatment.

☐ 0 ☐ 1 ☐ 2 ☐ 3

16. Outline your protocol for evaluating and addressing postoperative complications such as pulmonary embolism or deep vein thrombosis in patients undergoing esophageal surgery.

Answer1.1: If a patient undergoing esophageal surgery develops symptoms suggestive of pulmonary embolism or deep vein thrombosis (DVT), a thorough clinical assessment is conducted. This may include imaging studies such as CT pulmonary angiography or lower extremity ultrasound. Treatment typically involves anticoagulation therapy, with the choice of agent and duration determined by the

specific clinical scenario and risk factors.

Answer1.2: Prophylactic measures include anticoagulants, mechanical prophylaxis (e.g., compression devices), and early mobilization. Suspected cases of PE or DVT require immediate evaluation with Doppler ultrasonography or CT pulmonary angiography and treatment with anticoagulation.

Answer1.3: Prophylactic measures include early mobilization, compression stockings, and pharmacologic prophylaxis with anticoagulants. Should a PE or DVT occur, treatment involves anticoagulation therapy, with the duration and intensity based on the extent of the thrombosis and individual risk factors for bleeding.

☐ 0 ☐ 1 ☐ 2 ☐ 3

17. Share the recommended approach to the management of patients who experience recurrent or metastatic esophageal cancer following their initial surgical resection.

Answer1.1: In a patient with recurrent or metastatic esophageal cancer after initial surgical resection, treatment options depend on factors such as the location and extent of recurrence, prior treatments, and the patient's overall health. This may involve systemic therapy with chemotherapy or targeted agents, and in select cases, local interventions like radiation therapy or surgery for isolated metastases.

Answer1.2: Options include palliative chemotherapy, targeted therapy, radiation therapy, and supportive care measures. The management focuses on symptom control, maintaining quality of life, and considering the patient's overall health status and preferences.

Answer1.3: Options may include systemic chemotherapy, targeted therapy, immunotherapy, or palliative radiation for symptom control. The choice depends on the extent of recurrence, previous treatments, and patient performance status. Supportive care focusing on quality of life is paramount.

☐ 0 ☐ 1 ☐ 2 ☐ 3

18. How do you address nutritional support in the postoperative period for patients who have undergone esophageal surgery?

Answer1.1: After esophageal surgery, especially with gastric conduit reconstruction, a coordinated nutritional plan is crucial. This may involve early initiation of enteral feeding through a nasojejunal tube or gastrostomy tube to meet the patient's caloric and protein needs. Close monitoring of nutritional status and regular dietitian consultations are essential for optimizing recovery.

Answer1.2: Nutritional assessment and support are critical, involving oral, enteral, or parenteral nutrition based on the patient's ability to tolerate oral intake and nutritional needs. Dietary counseling and supplementation may be necessary to ensure adequate nutrition.

Answer1.3: Nutritional assessment and support are critical components of postoperative care. Early enteral nutrition through a jejunostomy tube, if placed, or

oral nutrition as tolerated. A dietitian's involvement for tailored dietary advice helps manage specific issues like dumping syndrome or malabsorption.

☐ 0 ☐ 1 ☐ 2 ☐ 3

19. Discuss the approaches to addressing postoperative gastroesophageal reflux in patients who have undergone esophagectomy.

Answer1.1: If a patient experiences postoperative gastroesophageal reflux after esophagectomy, management may involve lifestyle modifications (e.g., avoiding large meals close to bedtime), proton pump inhibitors, and potentially prokinetic agents. In some cases, revisional surgery or endoscopic interventions like anti-reflux procedures may be considered.

Answer1.2: Management includes lifestyle modifications, pharmacotherapy (e.g., proton pump inhibitors), and, in some cases, surgical interventions to correct anatomical contributors to reflux. Monitoring and adjusting treatment based on symptom control and patient tolerance are key.

Answer1.3: Management strategies include lifestyle modifications, elevation of the head of the bed, and pharmacologic treatment with proton pump inhibitors or H2 blockers. In severe cases, surgical interventions such as fundoplication may be considered if anatomically feasible.

☐ 0 ☐ 1 ☐ 2 ☐ 3

20. What is the target intraoperative blood loss volume, based on the patient's preoperative hemoglobin level and comorbidities, to minimize the risk of postoperative complications and ensure adequate tissue oxygenation during esophagectomy?

Answer1.1: Controlling intraoperative blood loss is crucial for minimizing postoperative complications and ensuring adequate tissue perfusion. The target blood loss volume depends on factors like preoperative hemoglobin levels, comorbidities, and surgical technique. For example, in a patient with significant comorbidities and low preoperative hemoglobin, stricter blood loss control measures may be necessary.
In a patient undergoing esophagectomy with a preoperative hemoglobin level of 10 g/dL and multiple comorbidities, the surgical team aims to limit blood loss to less than 500 mL. This approach helps prevent postoperative anemia and related complications.

Answer1.2: The target for intraoperative blood loss cannot be strictly defined as it varies with each patient's preoperative hemoglobin level, comorbidities, and the complexity of the surgical procedure. The goal is to minimize blood loss to reduce the risk of postoperative anemia and ensure adequate tissue oxygenation. Strategies include meticulous surgical technique, preoperative optimization of hemoglobin levels, use of minimally invasive surgical techniques, and intraoperative blood management protocols, including cell salvage and controlled hypotension when appropriate.

Answer1.3: The goal is to minimize blood loss to lessen postoperative complications

and maintain adequate tissue oxygenation. The target blood loss is not a fixed volume but is tailored to the patient's baseline hemoglobin and physiological reserve. Strategies to minimize blood loss include meticulous surgical technique, use of minimally invasive approaches, and intraoperative monitoring and management of coagulation status. Preoperative optimization of hemoglobin levels, especially in patients with anemia or comorbidities, is crucial to improve tolerance to blood loss.

☐ 0 ☐ 1 ☐ 2 ☐ 3

21. Elaborate on the role of minimally invasive techniques, such as robotic surgery, in esophageal procedures, and highlight the potential advantages over traditional open approaches.

Answer1.1: Consider a patient with early-stage esophageal cancer. Minimally invasive esophagectomy (MIE), including robotic-assisted approaches, may offer benefits such as reduced blood loss, shorter hospital stay, and faster recovery compared to traditional open surgery. This can lead to improved postoperative outcomes and a quicker return to normal activities.

Answer1.2: Minimally invasive techniques, such as laparoscopic and robotic-assisted surgery, have increasingly become the standard in esophageal procedures due to their significant advantages over traditional open surgery. These include reduced postoperative pain, shorter hospital stays, faster return to normal activities, and potentially lower rates of surgical complications. Robotic surgery offers enhanced dexterity, precision, and visualization for the surgeon, which is particularly beneficial in the complex anatomy of the esophagus. However, these techniques require specialized training and expertise.

Answer1.3: Minimally invasive techniques, including laparoscopic and robotic-assisted surgery, offer significant advantages over traditional open esophagectomy. These include reduced postoperative pain, shorter hospital stay, faster recovery, and potentially lower rates of respiratory complications. Robotic surgery provides enhanced dexterity, precision, and visualization, which may further reduce blood loss and nerve injury risk. However, the selection of patients for these approaches should consider tumor characteristics, patient anatomy, and the surgical team's expertise.

☐ 0 ☐ 1 ☐ 2 ☐ 3

22. Explain your approach to managing complications associated with conduit ischemia or necrosis following esophagectomy.

Answer1.1: In a patient who develops conduit ischemia or necrosis after esophagectomy, prompt intervention is crucial. This may involve re-exploration, assessment of vascular supply, and potentially revision of the anastomosis or conduit. In severe cases, resection of the compromised segment and reconstruction may be necessary.

Answer1.2: Conduit ischemia or necrosis following esophagectomy is a serious

complication requiring prompt recognition and management. Initial approaches may include optimizing hemodynamic status and nutritional support, with close monitoring for signs of systemic infection or sepsis. Endoscopic evaluation is essential for assessing the extent of ischemia or necrosis. Treatment options range from conservative management with antibiotics and nutritional support to surgical intervention for resection of the necrotic segment and reconstruction if necessary.

Answer1.3: Conduit ischemia or necrosis is a serious complication. Management begins with prevention, including careful surgical technique to preserve blood supply during conduit formation. Early detection is critical, using clinical signs or intraoperative technologies like fluorescence angiography. Mild cases of ischemia may be managed conservatively, but necrosis typically requires surgical intervention, such as conduit removal and reconstruction. Postoperative monitoring for signs of ischemia or necrosis is vital for early intervention.

☐ 0 ☐ 1 ☐ 2 ☐ 3

23. Discuss the pivotal factors in the surgical treatment of achalasia, and elaborate on how the selection of a procedure (e.g., Heller myotomy) influences outcomes.

Answer1.1: A patient with achalasia may be evaluated for surgical management. The primary intervention is a Heller myotomy, which involves dividing the muscles at the lower esophageal sphincter to allow for improved esophageal emptying. The choice of approach (laparoscopic or open) depends on factors like surgeon expertise and patient characteristics. Success rates and complication profiles are comparable between the two approaches.

Answer1.2: The choice of surgical procedure for achalasia, such as Heller myotomy, is influenced by factors like patient's symptom severity, esophageal motility, and anatomy. Heller myotomy, often performed laparoscopically, involves cutting the muscle fibers of the lower esophageal sphincter to relieve obstruction. Outcomes are generally favorable, with significant improvement in dysphagia and quality of life. The procedure may be combined with a partial fundoplication to prevent postoperative gastroesophageal reflux.

Answer1.3: The choice of surgical procedure, such as Heller myotomy versus peroral endoscopic myotomy (POEM), depends on patient factors including age, comorbidities, and preferences, as well as the disease's characteristics. Heller myotomy, often combined with partial fundoplication, is traditionally preferred for its durability and effectiveness in reducing dysphagia. POEM is a less invasive option with similar efficacy but may have a higher risk of postoperative gastroesophageal reflux. The selection of the procedure impacts outcomes, particularly in terms of symptom relief and quality of life.

☐ 0 ☐ 1 ☐ 2 ☐ 3

24. How do you approach patients with advanced esophageal cancer who are not candidates for curative surgery, and what are the palliative options available?

Answer1.1: In patients with advanced esophageal cancer who are not candidates for curative surgery, palliative measures are crucial. This may involve interventions like stent placement for relieving dysphagia, chemotherapy or radiation therapy to control symptoms and slow disease progression, and supportive care to address pain, nutrition, and quality of life.

Answer1.2: For patients not candidates for curative surgery, palliative care aims to improve quality of life and manage symptoms. Options include palliative chemotherapy or radiotherapy, endoscopic stent placement to relieve obstruction, nutritional support, and pain management. Palliative care services can also provide emotional and psychological support for patients and their families.

Answer1.3: For patients with advanced esophageal cancer not amenable to curative surgery, palliative care aims to improve quality of life and manage symptoms. Options include stenting for esophageal obstruction, radiation therapy for pain or bleeding control, and systemic therapies (chemotherapy, targeted therapy, immunotherapy) to manage disease symptoms and potentially extend life. Supportive care, including nutritional support and pain management, is also crucial.

☐ 0 ☐ 1 ☐ 2 ☐ 3

25. Outline the criteria for considering salvage surgery in instances of recurrent esophageal cancer following initial treatment.

Answer1.1: If a patient experiences recurrence of esophageal cancer after initial treatment with surgery or other modalities, salvage surgery may be considered in select cases. Indications for salvage surgery include isolated local recurrence that is amenable to surgical resection, absence of distant metastasis, and overall good performance status of the patient.

Answer1.2: Salvage surgery for recurrent esophageal cancer is considered based on factors like the location and extent of recurrence, the patient's overall health and performance status, and the absence of distant metastases. It's a complex decision-making process, often involving a multidisciplinary team, to evaluate the potential for achieving clear margins and the patient's ability to withstand another major surgery.

Answer1.3: Salvage surgery is considered for patients with localized recurrence after initial treatment without evidence of distant metastasis. Criteria include the feasibility of achieving clear surgical margins, the patient's overall health and ability to withstand surgery, and the absence of alternative, less invasive curative options. Multidisciplinary evaluation is essential to balance the potential benefits against the risks of morbidity and mortality.

☐ 0 ☐ 1 ☐ 2 ☐ 3

26. How do you assess and manage postoperative infections in patients who have undergone esophageal surgery?

Answer1.1: If a patient develops a postoperative infection after esophageal surgery, a

comprehensive evaluation is performed. This may involve obtaining cultures from relevant sites (e.g., wound, chest tube), imaging studies, and assessment of systemic signs of infection. Treatment typically involves targeted antibiotics based on culture sensitivities, along with drainage or surgical intervention if indicated.

Answer1.2: Postoperative infections are managed based on their nature and severity, involving antibiotics for surgical site or systemic infections, and interventions for abscess drainage or anastomotic leaks. Prevention strategies, including perioperative antibiotics, strict adherence to sterile technique, and early mobilization, are crucial. Monitoring for signs of infection is essential for early detection and treatment.

Answer1.3: Preventive strategies include perioperative antibiotics, sterile surgical technique, and careful monitoring. Postoperative infections are managed based on the type and severity, ranging from antibiotics for minor infections to surgical intervention for abscesses or deep infections. Early diagnosis and treatment are key to preventing sepsis and other complications.

☐ 0 ☐ 1 ☐ 2 ☐ 3

27. Elaborate on the long-term quality of life outcomes for patients following esophageal surgery, and delineate how these outcomes may differ based on the surgical approach and type of disease.

Answer1.1: Long-term quality of life outcomes for patients after esophageal surgery can vary based on factors like surgical approach (open vs. minimally invasive), type of reconstruction, and underlying disease. For example, patients with early-stage esophageal cancer who undergo minimally invasive surgery with gastric conduit reconstruction may experience better long-term quality of life compared to those with more advanced disease who require more extensive surgery.

Answer1.2: The long-term quality of life following esophageal surgery varies, with factors including the type of surgery, extent of disease, and occurrence of complications playing significant roles. Issues like dysphagia, nutritional challenges, and gastroesophageal reflux can impact quality of life. Multidisciplinary care, including dietary management, physical therapy, and psychological support, is key to addressing these concerns.

Answer1.3: Long-term outcomes vary by surgical approach, extent of surgery, and disease type. Factors affecting quality of life include nutritional status, ability to swallow, gastroesophageal reflux, and psychological well-being. Multidisciplinary care, including nutritional support, rehabilitation, and counseling, can help address these issues and improve overall quality of life.

☐ 0 ☐ 1 ☐ 2 ☐ 3

28. How do you address postoperative psychological and emotional well-being in patients who have undergone esophageal surgery, particularly those with a history of mental disorders?

Answer1.1: Patients who have undergone esophageal surgery, especially those with a

history of mental disorders, may require specialized psychological support. This could involve regular assessments by a mental health professional, psychoeducation, and potentially therapy or medication management. Collaborative care between surgical and mental health teams is essential.

Answer1.2: The psychological and emotional well-being of patients post-esophageal surgery is addressed through a comprehensive approach that includes psychological assessment, counseling, support groups, and, if necessary, psychiatric medications. Addressing mental health is integral to recovery, emphasizing the importance of screening for depression, anxiety, and other disorders pre- and postoperatively.

Answer1.3: Psychological support is integral to recovery, particularly for patients with pre-existing mental disorders. This includes counseling, support groups, and medication when necessary. Addressing emotional well-being is crucial for enhancing recovery, adherence to follow-up care, and overall quality of life.

☐ 0 ☐ 1 ☐ 2 ☐ 3

29. Outline the factors to be taken into account for preserving fertility in young patients undergoing esophageal surgery.

Answer1.1: In young patients undergoing esophageal surgery, fertility preservation is an important consideration, especially if they have not completed their family planning. Preoperative discussions regarding the potential impact of surgery on fertility, options for preservation (e.g., oocyte or sperm cryopreservation), and referral to reproductive specialists are important steps in the management plan.

Answer1.2: Factors to consider for preserving fertility include the potential effects of surgery and adjuvant therapy on reproductive organs and function. Options such as sperm banking for men and egg or embryo freezing for women should be discussed with patients desiring future fertility before treatment initiation.

Answer1.3: Fertility preservation should be discussed early in the treatment planning process. Options may include cryopreservation of sperm or oocytes, depending on the patient's age, desire for future fertility, and urgency of treatment. Collaboration with reproductive specialists is important for providing comprehensive care.

☐ 0 ☐ 1 ☐ 2 ☐ 3

30. Discuss the criteria used to assess the suitability of esophageal surgery for elderly patients with esophageal disease, and highlight the particular perioperative factors taken into account.

Answer1.1: In elderly patients with esophageal disease, the decision for surgery involves a comprehensive evaluation of their overall health, functional status, and goals of care. The presence of significant comorbidities, cognitive function, and frailty are important factors to consider. Additionally, perioperative considerations in the elderly may include tailored anesthesia, vigilant postoperative care, and comprehensive geriatric assessment to optimize outcomes.

Answer1.2: The suitability for esophageal surgery in elderly patients involves a

comprehensive assessment of their physiological age, comorbidities, functional status, and the expected quality of life post-surgery. Special attention is given to optimizing comorbid conditions, assessing nutritional status, and tailoring perioperative care to minimize risks and enhance recovery.

Answer1.3: Suitability for surgery in elderly patients is assessed through comprehensive evaluation of comorbidities, functional status, and life expectancy. Perioperative care focuses on minimizing complications, such as choosing minimally invasive approaches when appropriate, and tailored anesthesia and pain management strategies to enhance recovery.

☐ 0 ☐ 1 ☐ 2 ☐ 3

31. A 65-year-old male presents with a T1N0 adenocarcinoma at the gastroesophageal junction. What are the considerations for choosing between endoscopic resection and surgical intervention? How might patient factors influence this decision?

Answer1.1: In this case, the decision between endoscopic resection and surgical intervention depends on several factors. Endoscopic resection, such as endoscopic mucosal resection (EMR) or endoscopic submucosal dissection (ESD), may be suitable for early-stage, well-differentiated tumors limited to the mucosa. This offers a less invasive approach with lower morbidity. However, the tumor size, depth of invasion, and presence of high-risk features like lymphovascular invasion should be carefully evaluated. If the patient has a well-differentiated T1a adenocarcinoma with no high-risk features and is a poor surgical candidate due to significant comorbidities, endoscopic resection may be considered. Regular endoscopic surveillance is then crucial to monitor for any recurrence or residual disease.

Answer1.2: The decision between endoscopic resection and surgical intervention for a T1N0 adenocarcinoma at the gastroesophageal junction depends on tumor depth, size, and histologic features, as well as patient preferences, comorbidities, and overall health. Endoscopic resection may be appropriate for superficial, well-differentiated tumors without lymphovascular invasion, while surgery is indicated for deeper or poorly differentiated tumors.

Answer1.3: The choice between endoscopic resection and surgical intervention depends on the lesion's depth of invasion, differentiation, and lymphovascular involvement. Endoscopic resection is preferred for superficial lesions with low risk of lymph node metastasis. Surgical intervention is considered for deeper lesions or when high-risk features are present. Patient factors, including comorbidities and preferences, also influence the decision.

☐ 0 ☐ 1 ☐ 2 ☐ 3

32. A 50-year-old female with longstanding achalasia has failed conservative treatment. What are the advantages and potential complications of different

surgical approaches for treating achalasia? How do you select the most appropriate technique for this patient?

Answer1.1: For a patient with achalasia who has failed conservative treatment, surgical options like laparoscopic Heller myotomy (LHM) or peroral endoscopic myotomy (POEM) should be considered. LHM involves laparoscopic division of the lower esophageal sphincter, while POEM is an endoscopic approach. The choice depends on factors such as the patient's anatomy, surgeon expertise, and patient preference. If the patient has a history of prior abdominal surgeries, making laparoscopic access challenging, and has a favorable anatomy for endoscopy, POEM may be the preferred option.

Answer1.2: For patients with achalasia who have failed conservative treatment, surgical options like Heller myotomy provide significant symptom relief. The choice of technique is guided by patient factors and surgeon expertise. Potential complications include perforation and postoperative reflux, managed with partial fundoplication. The selection of the most appropriate technique considers patient anatomy, esophageal function, and the presence of comorbidities.

Answer1.3: Surgical options include Heller myotomy and POEM, with both aiming to relieve dysphagia by disrupting the lower esophageal sphincter. Heller myotomy is traditionally performed with a partial fundoplication to prevent reflux, while POEM offers a less invasive alternative without abdominal incisions. Selection depends on patient-specific factors, including anatomy and preference, as well as the surgeon's expertise.

☐ 0 ☐ 1 ☐ 2 ☐ 3

33. A 60-year-old male with a history of esophagectomy for cancer now presents with recurrent dysphagia. What diagnostic evaluations and interventions would you consider to evaluate the cause of his symptoms?

Answer1.1: Recurrent dysphagia post-esophagectomy warrants a thorough evaluation. This may involve upper endoscopy with dilation or stent placement to address any anastomotic strictures or other obstructions. Imaging studies like a barium swallow or CT scan can help identify any structural issues. If endoscopy reveals an anastomotic stricture, it can be managed with dilation. If the patient continues to have dysphagia despite dilation, further evaluation with imaging may be necessary to identify any other contributing factors.

Answer1.2: Diagnostic evaluations for recurrent dysphagia post-esophagectomy include endoscopy, barium swallow study, and esophageal manometry to assess for stricture, recurrence of cancer, or motility disorders. Intervention depends on the underlying cause, with treatments ranging from endoscopic dilation for strictures to more complex interventions for recurrent disease.

Answer1.3: Diagnostic evaluation should include endoscopy to assess for stricture or recurrence, barium swallow study to evaluate esophageal morphology, and manometry if motility disorder is suspected. Treatment depends on the underlying cause, ranging from endoscopic dilation for strictures to more complex interventions

for recurrence or motility issues.

☐ 0 ☐ 1 ☐ 2 ☐ 3

34. A 70-year-old patient develops a chyle leak post-esophagectomy. What are the immediate steps for management, and what are the potential long-term consequences of this complication?

Answer1.1: In the case of a chyle leak post-esophagectomy, immediate management involves drainage of the chyle collection, dietary modifications (low-fat diet or medium-chain triglyceride supplementation), and potentially somatostatin analogs to reduce chyle production. If the patient's chyle leak persists despite conservative measures, interventional radiology-guided thoracic duct embolization may be considered to address the source of the leak.

Answer1.2: Immediate management includes dietary modifications to reduce chylous output, parenteral nutrition to support nutritional status, and octreotide to reduce chyle production. Surgical intervention may be required for persistent leaks. Long-term consequences can include nutritional deficiencies and immunosuppression, necessitating close monitoring and management.

Answer1.3: Initial management includes dietary modification to a medium-chain triglyceride diet or total parenteral nutrition, along with octreotide to reduce chyle flow. If conservative measures fail, surgical intervention to ligate the leaking lymphatic vessels may be necessary. Untreated chyle leak can lead to malnutrition, immunodeficiency, and increased mortality.

☐ 0 ☐ 1 ☐ 2 ☐ 3

35. 55-year-old male develops an anastomotic stricture following esophagectomy. How would you approach the treatment of this stricture, and what are the considerations for long-term management?

Answer1.1: An anastomotic stricture after esophagectomy may require endoscopic dilation using bougie dilators or controlled radial expansion balloons. In refractory cases, stenting or endoscopic incisional therapy may be considered. If a patient develops a symptomatic stricture after esophagectomy, initial treatment with endoscopic dilation is typically performed. In cases of severe or refractory strictures, stenting may be used as a temporizing measure.

Answer1.2: Treatment involves endoscopic dilation, with balloon or bougie dilators, and may require multiple sessions to achieve sustained improvement. For refractory strictures, steroid injections or temporary stent placement may be considered. Long-term management focuses on monitoring symptoms and nutritional status, with repeated interventions as necessary.

Answer1.3: The primary treatment for anastomotic strictures is endoscopic dilation, often requiring multiple sessions. In cases of refractory strictures, steroid injections, application of mitomycin C, or stent placement may be considered. Severe or recurrent cases might require surgical revision. Long-term management focuses on

monitoring for recurrence and maintaining nutritional status.

☐ 0 ☐ 1 ☐ 2 ☐ 3
